# Supplementary material for: The Relationship between Clock Genes, Sirtuin 1, and Mitochondrial Activity in Head and Neck Squamous Cell Cancer: Effects of Melatonin Treatment
Source: Int J Mol Sci. 2023 Oct 9;24(19):15030. doi: 10.3390/ijms241915030 (PMC10573844; doi:10.3390/ijms241915030)
Supplement: Supplementary file 1 [file ijms-24-15030-s001.zip › ijms-2634362-supplementary.pdf]

SUPPLEMENTARY FILES (FIGURES AND TABLES)

Figure S1

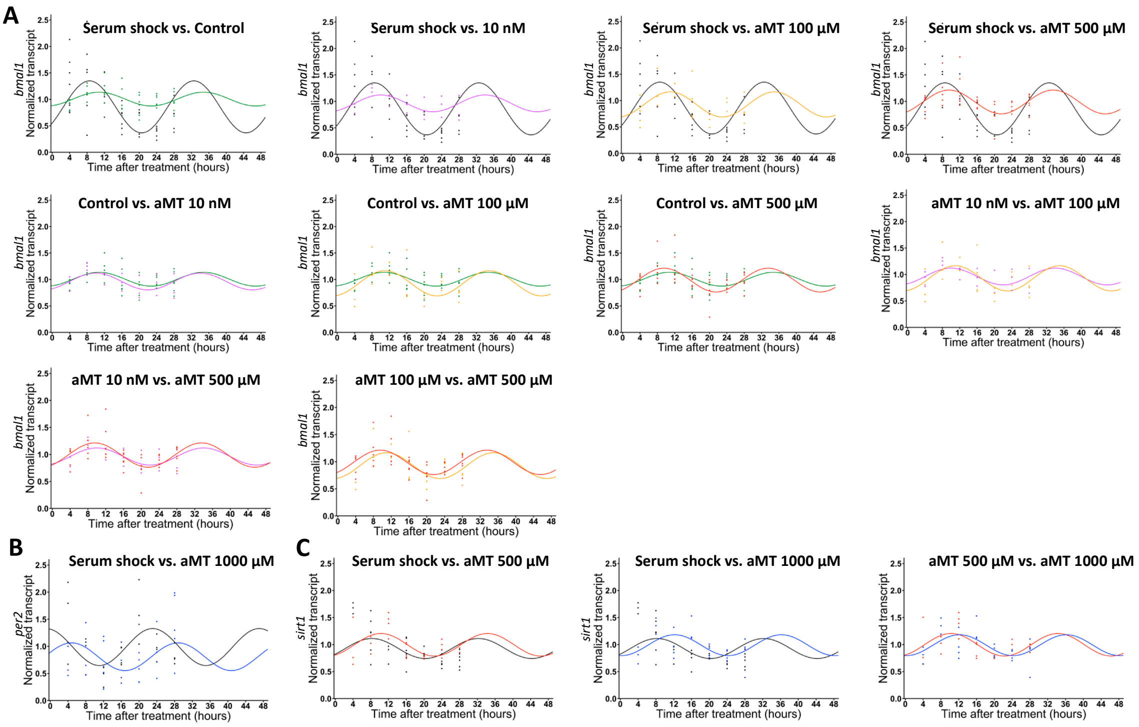

Figure S2

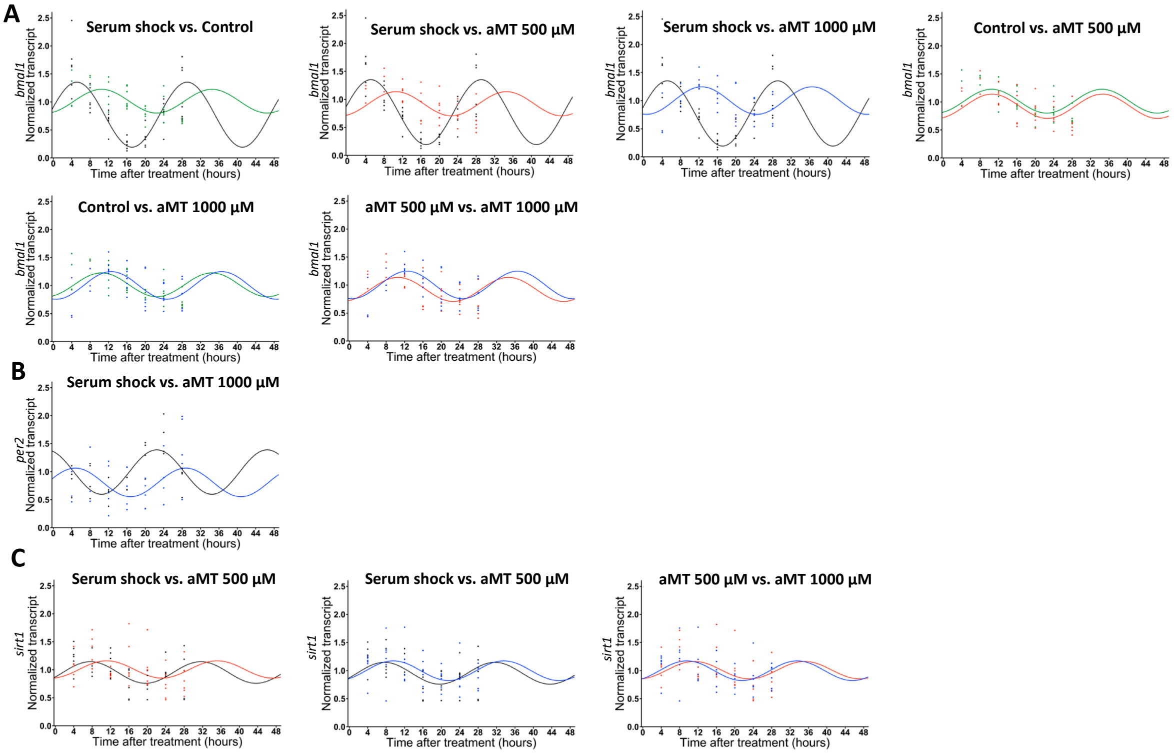

Table S1

Treatment	Presence of rhythmicity (p-value)	Acrophase	Amplitude
aMT 10 nM	<0.05	9.981817	0.158323
aMT 100 µM	<0.05	10.95637	0.238408

Table S2

Comparison	P-value for acrophase difference	P-value for amplitude difference
Serum shock vs. aMT 10 nM	ns (0.614919)	<0.05 (0.037591)
Serum shock vs. aMT 100 µM	ns (0.198786)	ns (0.119811)
Control vs. aMT 10 nM	ns (0.657939)	ns (0.718146)
Control vs. aMT 100 µM	ns (0.908333)	ns (0.241201)
aMT 10 nM vs. aMT 100 µM	ns (0.6226)	ns (0.474727)
aMT 10 nM vs. aMT 500 µM	ns (0.783253)	ns (0.445071)
aMT 100 µM vs. aMT 500 µM	ns (0.299178)	ns (0.898303)

Figure S3

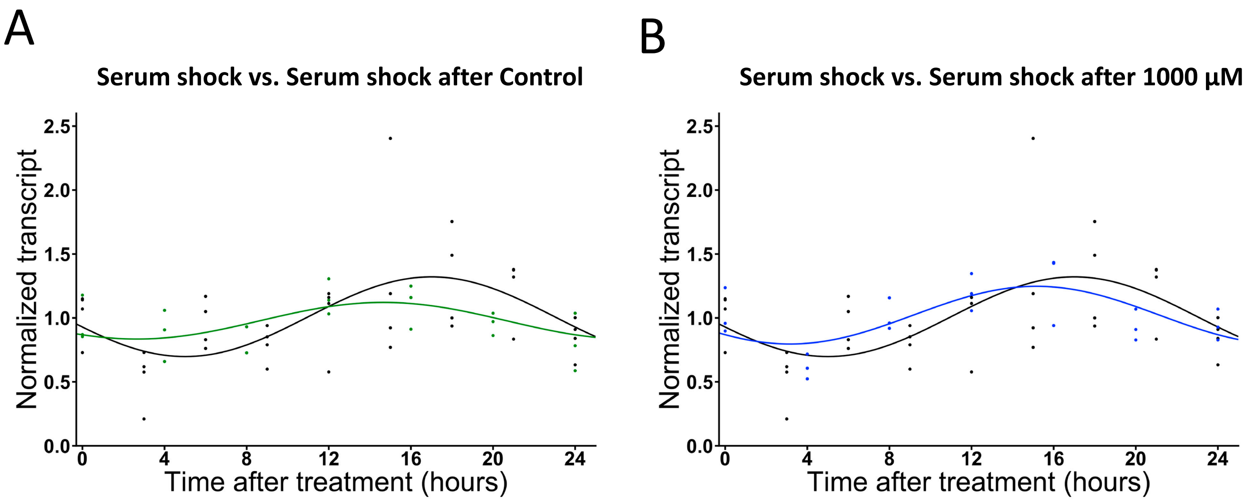

LEGENDS FOR SUPPLEMENTARY FIGURES

Figure S1. Cal-27 Cosinor's best fits comparison of gene expression between different experimental groups. (**A**) *bmal1*; (**B**) *per2*; (**C**) *sirt1*. Serum shock (black), control (green) and aMT 10 nM (purple), 100 µM (orange), 500 µM (red) and 1000 µM (blue) treatments; n= 3-6 independent experiments.

Figure S2. SCC9 Cosinor's best fits comparison of gene expression between different experimental groups. (**A**) *bmal1*; (**B**) *per2*; (**C**) *sirt1*. Serum shock (black), control (green) and aMT 500 µM (red) and 1000 µM (blue) treatments; n= 3-6 independent experiments.

Table S1. Cosinor analysis of relative expression of the clock gene Bmal1 in HNSCC cell line Cal-27 after aMT treatments (10 nM and 100 µM).

Table S2. Circadian rhythm comparison of relative expression of the clock genes Bmal1 in HNSCC cell line Cal-27. * $p<0.05$.

Figure S3. Cosinor's best fits comparison of OCR between different experimental groups. (A) Serum shock treatment (black) *versus* serum shock after control treatment (green); (B) serum shock treatment (black) *versus* serum shock after melatonin 1000 µM treatment (blue); n= 3-6 independent experiments.
